# Assessment of Surrogate Markers for Cardiovascular Disease in Familial Mediterranean Fever-Related Amyloidosis Patients Homozygous for M694V Mutation in *MEFV* Gene

**DOI:** 10.3390/life12050631

**Published:** 2022-04-25

**Authors:** Sezgin Sahin, Micol Romano, Ferhat Guzel, David Piskin, Dimitri Poddighe, Siren Sezer, Ozgur Kasapcopur, C. Thomas Appleton, Ilker Yilmaz, Erkan Demirkaya

**Affiliations:** 1 Department of Paediatric Rheumatology, Istanbul University-Cerrahpasa, Istanbul 34098, Turkey; ozgurkasapcopur@hotmail.com; 2 Division of Paediatric Rheumatology, Department of Paediatrics, Schulich School of Medicine & Dentistry, University of Western Ontario, London, ON N6A 5C1, Canada; micol.dr.romano@gmail.com (M.R.); erkan.demirkaya@uwo.ca (E.D.); 3 Canadian Behcet and Autoinflammatory Disease Center (CAN-BE-AID), University of Western Ontario, London, ON N6A 5C1, Canada; tom.appleton@sjhc.london.on.ca; 4 Molecular Genetics Laboratories, Genetics Research and Genome Center, Department of Research and Development, Ant Biotechnology, Istanbul 34775, Turkey; ferhat@gentera.com.tr; 5 Lawson Health Research Institute, London Health Sciences Center, London, ON N6C 2R5, Canada; david.piskin@lhsc.on.ca; 6 Department of Epidemiology and Biostatistics, Schulich School of Medicine & Dentistry, University of Western Ontario, London, ON N6A 5C1, Canada; 7 Department of Medicine, Nazarbayev University School of Medicine, Nur-Sultan 010000, Kazakhstan; dimitri.poddighe@nu.edu.kz; 8 Clinical Academic Department of Pediatrics, National Research Center of Maternal and Child Health, University Medical Center, Nur-Sultan 010000, Kazakhstan; 9 Division of Nephrology, Atilim University Faculty of Medicine, Ankara 06830, Turkey; sirensezer@hotmail.com; 10 Division of Rheumatology, Department of Medicine, Schulich School of Medicine and Dentistry, University of Western Ontario, London, ON N6A 5C1, Canada; 11 Department of Physiology and Pharmacology, Schulich School of Medicine and Dentistry, University of Western Ontario, London, ON N6A 5C1, Canada; 12 Epigenetic Health Solutions, Unit of Nephrology, Ankara 06810, Turkey; mahmutilkeryilmaz@gmail.com

**Keywords:** familial Mediterranean fever, M694V homozygosity, AA amyloidosis, cardiovascular disease, flow-mediated dilatation, carotid artery intima-media thickness

## Abstract

Cardiovascular disease (CVD) remains underestimated in familial Mediterranean fever-associated AA amyloidosis (FMF-AA). We aimed to compare early markers of endothelial dysfunction and atherosclerosis in FMF-AA with a homozygous M694V mutation (Group 1 = 76 patients) in the Mediterranean fever (*MEFV*) gene and in patients with other genotypes (Group 2 = 93 patients). Measures of increased risk for future CVD events and endothelial dysfunction, including flow-mediated dilatation (FMD), pentraxin-3 (PTX3), and carotid intima-media thickness (cIMT), and fibroblast growth factor 23 (FGF23) as a marker of atherosclerotic vascular disease were compared between groups. The frequency of clinical FMF manifestations did not differ between the two groups apart from arthritis (76.3% in Group 1 and 59.1% in Group 2, *p* < 0.05). FMD was significantly lower in Group 1 when compared with Group 2 (MD [95% CI]: −0.6 [(−0.89)–(−0.31)]). cIMT, FGF23, and PTX3 levels were higher in Group 1 (cIMT MD [95% CI]: 0.12 [0.08–0.16]; FGF23 MD [95% CI]: 12.8 [5.9–19.6]; PTX3 MD [95% CI]: 13.3 [8.9–17.5]). In patients with FMF-AA, M694V homozygosity is associated with lower FMD values and higher cIMT, FGF23, and PTX3 levels, suggesting increased CVD risk profiles. These data suggest that a genotype–phenotype association exists in terms of endothelial dysfunction and atherosclerosis in patients with FMF-AA.

## 1. Introduction

Familial Mediterranean fever (FMF) is the most frequent hereditary autoinflammatory disease globally and is mainly observed among eastern Mediterranean populations, including people of Turkish, Armenian, non-Ashkenazi Jewish, Italian, and Arabic origins [1,2,3]. FMF is characterized by periodic episodes of inflammation with fever, polyserositis, and occasionally with recurrent non-erosive monoarthritis or erysipelas-like erythema, all of which are due to autosomal recessive mutations of the Mediterranean fever (*MEFV*) gene [4]. Colchicine is the mainstay of the treatment for this lifelong disease to control these remitting-relapsing episodes and thereby to prevent the most devastating and fatal complication of FMF, namely systemic AA amyloidosis [5]. IL-1 inhibitors, including anakinra and canakinumab, have been safely and effectively used in colchicine-resistant or -intolerant FMF patients, accounting for 10–20% of all FMF patients [6,7,8,9]. 

Of note, the *MEFV* gene mutations in exon 10 are high-penetrance mutations which have been significantly associated with a more severe clinical course, higher rates of AA amyloidosis, more resistance to colchicine, and increased requirements for biologic agents [2,8,10,11,12,13,14]. The most frequent and well-recognized among these genotypes is the M694V allele, which has been identified as the most deleterious in a homozygous state [10,12]. The homozygous M694V variant has repeatedly been shown to be significantly and independently associated with renal amyloidosis in previous studies [10,12]. Moreover, higher levels of the IL-1 family cytokine IL-18, myeloid cell-derived S100A12, and caspase-1 have been reported to be released from the neutrophils of homozygous M694V patients compared to those FMF patients with other genotypes and healthy controls [13].

FMF is associated with increased all-cause mortality, of which 35% to 60% is due to renal amyloidosis [15]. However, cardiovascular diseases (CVDs) should not be underestimated in FMF patients, as they are still the leading cause of death globally [16]. Increased pro-inflammatory and/or inflammatory patterns in rheumatic diseases may cause a higher risk of CVD events with the concept of “accelerated atherosclerosis”. Raised risks of symptomatic CVD events in patients with rheumatoid arthritis (RA) and systemic lupus erythematosus (SLE) have been shown in a recently published meta-analysis [17]. As inflammatory diseases increase the risk of CVD, traditional CVD risk factors should be more rigorously identified and controlled in subjects with autoimmune and inflammatory diseases, especially those with active inflammation and higher mortality rates such as FMF. However, whether there is an increased risk for coronary artery disease (CAD) in FMF patients is a subject of some debate [18,19]. In a retrospective study in the Sicilian population, harboring the M694V allele increased the risk of developing myocardial infarction [11]. On the contrary, the unexpected lower CAD rates among FMF patients in some previous reports could be attributable to colchicine usage, since this alkaloid has recently emerged as a promising option for the prevention of atherosclerosis by controlling inflammation [20]. Other confounders such as earlier death of FMF patients from other causes related to FMF and small patient sample sizes may also be responsible for the lower CAD rates in these studies. Accordingly, the association between FMF and CAD is yet to be clarified. 

Measurement of intima-media thickness (IMT) on carotid ultrasound to detect the presence of any subclinical atherosclerosis may provide substantial clues for the elucidation of individual CVD risk. Both the American Heart Association/American College of Cardiology (AHA/ACC) and the European Society of Hypertension/European Society of Cardiology recommend measurement of carotid IMT (cIMT) as a class IIa recommendation with level of evidence B, even for asymptomatic elderly individuals or adults with traditional risk factors [21,22]. 

Flow-mediated dilatation (FMD) refers to a non-invasive ultrasound-mediated assessment of brachial artery dilatation following 5 min of forearm ischemia by an inflated cuff. Some evidence suggests that the reduced ability to change the diameter of the brachial artery is an indirect marker of an abnormally functioning endothelium [23,24].

Pentraxin-3 (PTX3), primarily secreted from endothelial cells, is a widely recognized biomarker of vascular endothelial injury. The rapid elevations in plasma levels of PTX3 in acute myocardial infarction (AMI), cardiac arrest, and heart failure have been addressed in a substantial number of studies, reflecting endothelial and tissue damage [25]. 

Fibroblast growth factor 23 (FGF23), a hormone produced by bone, is mainly responsible for vitamin D and phosphate homeostasis [26,27]. Elevated FGF23 levels have also been associated with several forms of adverse cardiovascular outcomes, including subclinical atherosclerotic disease, hypertension, left ventricular hypertrophy, and other cardiovascular events [26]. Whether or not a direct causality exists between elevated FGF23 levels and subclinical atherosclerosis is a subject of some debate. FGF23 has been found to directly cause phosphate-induced vascular calcification, whereas another hypothesis suggests that elevated FGF23 levels could be associated with atherosclerosis through hypertension [26,27].

There are limited data in FMF patients regarding vascular abnormalities, including atherosclerosis and endothelial dysfunction, two of the main contributors to CVD risk. Furthermore, risk assessments for future CVD events have not yet been evaluated between FMF amyloidosis patients with different genotypes. Herein, we aimed to assess the effect of M694V homozygosity on the early markers of CVD (FMD and PTX3 for endothelial dysfunction and cIMT and FGF23 for atherosclerosis) in patients with FMF-related amyloidosis.

## 2. Materials and Methods

### 2.1. Study Design and Patients

In this cross-sectional study, FMF patients complicated with amyloidosis (AA amyloidosis) were enrolled to the study from a comprehensive registry of patients with several underlying chronic renal diseases at the renal unit of the Epigenetic Health Center Outpatient Clinics and Gulhane School of Medicine in Turkey [28,29,30]. Patients were assigned to one of the following 2 groups according to the type of mutation in the *MEFV* gene: Group 1 (*n* = 76), patients homozygous for M694V; and Group 2 (*n* = 93), patients homozygous (other than M694V) or compound heterozygous (including M694V) for other *MEFV* variants. Informed consent was provided by each participant, and the study protocol was approved by the local ethical committee (GMMA (50687469-1491-70)). The study was performed according to the principles of the Declaration of Helsinki. All FMF amyloidosis patients without some traditional factors such as smoking, clinical history of CVD, overt diabetes mellitus, obesity (BMI > 30 kg/m^2^), and untreated hypertension that were referred between September 2003 and February 2020 were recruited.

All included subjects were evaluated by physical examination. Data on clinical manifestations (Table 1) and treatment regimens were documented (face-to-face interviews during the enrolment) retrospectively and prospectively. All patients were assessed for kidney function, *MEFV* mutations for genotyping, and histopathological diagnosis of amyloidosis. Tel Hashomer criteria were used for the diagnosis of FMF [4,31]. Amyloidosis was confirmed in all FMF patients with a positive staining pattern of kidney biopsy materials with Congo red dye. All patients were on colchicine treatment prior to the study period. Some of the patients on colchicine therapy were also using IL-1 inhibitors. All blood samples were collected, and measurements of cIMT and FMD were performed during the same study visit, when there had been no active attack (fever and/or polyserositis and/or arthritis and/or erysipelas-like erythema) for 2 weeks prior to the visit.

### 2.2. Laboratory Measurements

Blood sampling was performed after overnight fasting and the following laboratory parameters were recorded: fasting plasma glucose (FPG), total protein, serum albumin, calcium, phosphorus, total cholesterol, HDL cholesterol, LDL cholesterol, triglycerides, serum basal insulin, and high-sensitivity C-reactive protein levels in serum (hsCRP). Measurement of 24-hour urinary protein was determined by a turbidimetric test using trichloroacetic acid (TCA). Renal function was calculated by the modified Modification of Diet in Renal Disease (MDRD) formula in mL/min and expressed per 1.73 m^2^ of body surface area [32]. Homeostasis model assessment (HOMA) was calculated as: Homeostasis model assessment-insulin resistance (HOMA-IR) = FPG (mg/dL) × immunoreactive insulin (IRI) (µIU/mL)/405 [33]. The average of two reads was used for each measurement for all the samples.

High-performance liquid chromatography kits (ImmuChrom GmbH, Heppenheim, Germany) were used to measure 25-hydroxyvitamin D (25OHD). Parathyroid hormone (PTH) was measured by immunoradiometric assay using a reagent (Immulite Intact PTH) from Diagnostic Product Corporation (Los Angeles, CA, USA).

Serum FGF23 was determined using an intact FGF23 enzyme-linked immunosorbent assay (ELISA) in line with the instruction manual (Kainos Laboratories, Tokyo, Japan). The overall intra- and inter-assay coefficients of variation were 2.5% and 2.8%, respectively. 

Plasma levels of PTX3 were measured using a commercially available ELISA kit according to the manufacturer’s instructions (Perseus Proteomics). Both the intra- and inter-assay coefficients of variations were 5.0% with an ELISA system detection limit of 0.1 ng/mL. 

### 2.3. Endothelial and Vascular Assessment 

#### 2.3.1. Flow-Mediated Dilatation (FMD)

Systolic and diastolic blood pressure values were measured three times by a clinician after a resting period of 15 min in the morning, and mean values were calculated and recorded. The endothelial-dependent FMF was measured according to the criteria of the International Brachial Artery Reactivity Task Force [34]. The same principles as Celermajer et al. were used to evaluate endothelial dysfunction (ED) [23]. Prior to the examination, subjects were requested to stay in the supine position for 15 min. A single observer performed the measurements of the brachial artery 2–4 cm above the antecubital fossa using an ATL 5000 ultrasound system (Advanced Technology Laboratories Inc., Bothell, WA, USA) with a 12 MHz probe. 

Inflation of the pneumatic tourniquet to 200 mmHg for 5 min resulted in the disappearance of the radial pulse. Then, FMD measurements were made 1 min after deflation. Three consecutive measurements at the end-diastolic phase were recorded, and these ultrasound images were saved for later blinded analysis. The averages of these three diameters were calculated. The change in diameter in percentage compared with the baseline resting diameter was accepted as FMD. The intra-observer coefficient of variation was 5.1%.

#### 2.3.2. Carotid Artery Intima-Media Thickness (cIMT)

For measurement of c-IMT on both sides, high-resolution Doppler ultrasound (ATL 5000) with a 5–12 MHz linear transducer was used. Two consecutive longitudinal images of each common carotid artery were acquired 1–2 cm proximal to the bifurcation and then stored. The mean c-IMT was calculated by averaging the four measurements.

#### 2.3.3. Genetic Screening

The protocol for genetic screening was described in our previous work in detail [35]. A QIAamp DNA mini kit (Qiagen, Germany) was used for DNA extraction from blood samples according to standard procedures. The reverse-hybridization method (Vienna Lab Diagnostics, Vienna, Austria) was performed for mutation analysis [36]. Twelve variants in the *MEFV* gene were genotyped, including E148Q, P369S, F479L, M680I (G > C, G > A), I692 del, M694V, M694I, K695R, V726A, A744S, and R761H.

### 2.4. Statistical Analysis

In this cross-sectional study, descriptive statistics are presented as frequencies and percentages for categorical variables and median (minimum–maximum) for continuous data. The distribution of variables was assessed with graphical methods and the Kolmogorov–Smirnov test. Categorical variables were compared with the chi-square test and Fisher’s exact test where appropriate. The independent sample t-test and Mann–Whitney U test was used to compare continuous variables between groups. Pearson correlations were used to assess correlations between relevant parameters. Statistical analysis was performed using Statistical Package of Social Science (SPSS) for Windows, version 15.0 (SPSS Inc, Chicago, IL, USA). Mean differences and 95% confidence intervals were calculated, and a *p*-value < 0.05 was considered as statistically significant.

## 3. Results

**Figure 1:**
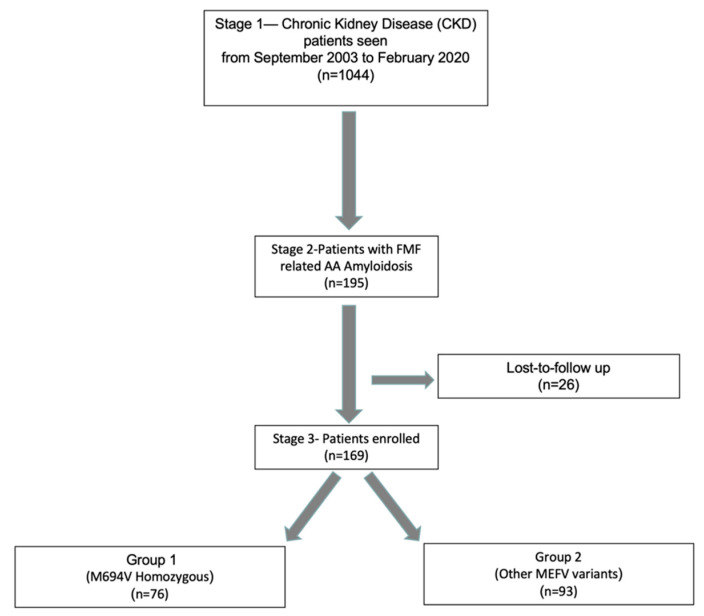
Flowchart of the registry and included patients.

**Figure 2:**
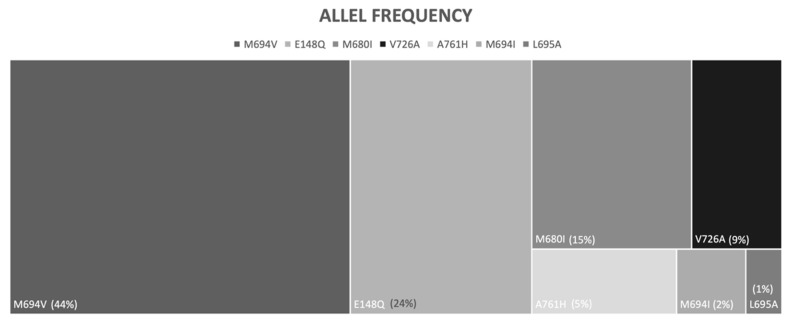
*MEFV* allele frequency in patients with AA amyloidosis secondary to FMF.

Our registry consisted of 195 patients with a diagnosis of AA amyloidosis. A total of 169 eligible amyloidosis patients secondary to FMF were enrolled to this study (Figure 1). Patients were assigned to one of the following two groups according to the mutation type in the *MEFV* gene: Group 1 (*n* = 76), patients homozygous for M694V (which is the most common genotype associated with the most severe clinical phenotype in FMF), and Group 2 (*n* = 93), patients homozygous (other than M694V) or compound heterozygous (including M694V) for other *MEFV* variants (Figure 2). 

Demographic and clinical features and the non-invasive markers of endothelial dysfunction (FMD and PTX3) and atherosclerosis (cIMT and FGF23) were compared between FMF-related AA amyloidosis patients in Group 1 and Group 2. Patients in Group 1 did not differ from those in Group 2 regarding age of diagnosis (either FMF or amyloidosis), gender distribution (Table 1). Characteristics of FMF episodes including fever, serositis, and erysipelas-like erythema were similar in the two groups, except for arthritis (76.3% in Group 1 and 59.1% in Group 2, *p* < 0.05) (Table 1). 

There were 76 patients in Group 1, all of whom were homozygous for M694V. Among the 93 patients in Group 2, the most frequently observed genotype was M694V/E148Q (*n* = 25), followed by E148Q/E148Q (*n* = 19), M680I/M680I (*n* = 9), M694V/M680I (*n* = 9), and V726A/V726A (*n* = 6). The number of patients who had ever used anakinra or canakinumab was not different between Group 1 (anakinra: *n* = 8 [10.5%], canakinumab: *n* = 9 [11.8%]) and Group 2 (anakinra: *n* = 16 [17.2%], canakinumab: *n* = 16 [17.2%]).

There was no significant difference between Group 1 and Group 2 in average blood pressure measurements, BMI, HOMA-IR levels, serum levels of hsCRP, total cholesterol, triglyceride, LDL and HDL cholesterol, glucose, insulin, PTH, and 25OHD (Table 2).

The 24-hour proteinuria level was higher in Group 1 than in Group 2 (mean difference [95% CI]: 3070.9 [2230.9–3910.9]). Brachial artery FMD was significantly lower in Group 1 when compared with Group 2 subjects (mean difference [95% CI]: −0.6 [(−0.89)–(−0.31)]). FGF23 and PTX3 levels were higher in Group 1 compared to Group 2 (FGF23 mean difference [95% CI]: 12.8 [5.9–19.6]; PTX3 mean difference [95% CI]: 13.3 [8.9–17.5]). cIMT values were higher in M694V homozygote patients (Group 1) than in patients with compound heterozygous (including M694V) for any *MEFV* variant (Group 2) (mean difference [95% CI]: 0.12 [0.08–0.16]) (Table 3).

In Group 1, there was a moderate negative correlation between FMD and CIMT (r = −0.44, *p* < 0.001), FGF23 (r = −0.48, *p* < 0.001), PTX3 (r = −0.48, *p* < 0.001), and proteinuria (r = −0.62, *p* < 0.001). Additionally, there was a moderate positive correlation between CIMT and FGF23 (r = 0.40, *p* < 0.001), PTX3 (r = 0.61, *p* < 0.001), and proteinuria (r = 0.52, *p* < 0.001). In Group 2, while there was a moderate negative correlation between FMD and proteinuria (r = −0.46, *p* < 0.001), there was a weak negative correlation between FMD and FGF23 (r = −0.31, *p* = 0.002). The correlation was moderately positive between CIMT and PTX3 (r = 0.43, *p* < 0.001), whereas it was weakly positive between CIMT and FGF23 (r = 0.22, *p* = 0.04) (Table 4).

## 4. Discussion

Our study demonstrated that M694V homozygosity is associated with lower FMD values and higher carotid intima-media thickness measurements in patients with FMF-related AA amyloidosis, indicating an increased CVD risk profile associated with an M694V homozygous genotype relative to compound heterozygous genotypes. Supporting this finding, patients with a homozygous M694V genotype had higher FGF23 and PTX3 levels, which are surrogate markers for atherosclerosis and endothelial dysfunction, respectively. Herein, a significant association was found between surrogate measures of CVD risk and the M694V allele, which has also been recognized as the most significant and frequent variant associated with a more severe clinical FMF phenotype. Together, these results support the hypothesis that FMF may be associated with increased CVD risk, at least in patients with AA amyloidosis. 

The risk of CVD has been demonstrated to be higher in chronic rheumatic diseases, including ankylosing spondylitis, psoriatic arthritis, and rheumatoid arthritis, than in the general population in a significant number of studies [37,38,39]. However, whether or not there is an increased risk for CVD in FMF patients is a matter of some dispute, since there are limited data on the risk of CVD in patients with FMF and also in patients with AA amyloidosis secondary to FMF and other autoinflammatory diseases [18,19,40]. In a recent study, mortality related to CVD was found to be 2.8-fold higher in patients with FMF-amyloidosis than in patients with other chronic kidney diseases [40]. Furthermore, lower FMD values and higher FGF23 and hsCRP levels were found to be associated with a higher risk of future CVD events in that recent study. 

Secondary AA amyloidosis is a devastating and life-threatening long-term complication of under-/untreated chronic inflammatory rheumatic diseases, autoinflammatory diseases, inflammatory bowel diseases, and chronic infections [41,42]. Importantly, early and aggressive treatment to suppress chronic inflammation can reduce the risk of AA amyloidosis. Similarly, CVD events have been described as the cause of death in 21% of the non-survivors in a retrospective study including AA amyloidosis patients secondary to long-standing, poorly controlled rheumatoid arthritis [43]. Abnormal vascular morphology and endothelial dysfunction have been demonstrated by measurement of cIMT and FMD in both primary and secondary amyloidosis in two previous studies [44,45]. In addition to atherosclerosis, vascular deposition of amyloid protein has been postulated as an underlying factor for these abnormal results. Likewise, higher cIMT but lower FMD values were also found in FMF patients without amyloidosis in a previous study, although it was limited by the low number of patients [46]. Uncontrolled persistent inflammation, which is the leading contributing factor for AA amyloidosis, is also a risk factor for CVD via atherosclerosis. Thus, prevention of episodes and alleviation of the overt or subclinical inflammation in FMF by colchicine or IL-1 inhibitors not only prevents the development of amyloidosis but could also thus, hypothetically, reduce the risk of atherosclerotic CVD. Regardless of genotype, a similar number of patients required anti-IL-1 therapy with anakinra or canakinumab, reducing the likelihood of confounding due to therapy in our study. However, proteinuria was higher in the homozygous M694V genotype group, which may or may not contribute to confounding due to the association of renal dysfunction with CVD. Considering the average age of the patients included in this study of 36 years, our findings further suggest a role of the M694V homozygous genotype in determining CVD risk. This study underscores other reports that suggest the existence of cardiovascular dysfunction in patients with FMF-related amyloidosis [40]. Since the early stages of atherosclerosis and endothelial dysfunction are thought to be reversible with aggressive medical intervention, periodic clinical CVD risk assessment should be considered in patients with FMF-related amyloidosis and possibly all patients with FMF as an at-risk population. However, aside from aggressive control of inflammation and traditional CVD risk factor assessments, current evidence is not available to guide the frequency or assessment methods that should be used in patients with FMF. Screening of these patients with the non-invasive surrogate markers as in our study may be beneficial before the establishment of structural changes [47]. 

Genotype–phenotype studies almost invariably revealed that M694V mutations are associated with a more severe clinical and molecular phenotype with a higher incidence of attacks, amyloidosis, and higher amounts of cytokine release [13,48,49]. Our results on the higher risk of endothelial dysfunction and atherosclerosis, which are early markers of CVD, and higher mortality rates in M694V homozygotes are partly in line with the results of a retrospective study in a Sicilian population. In terms of CVD events, the expression of the M694V allele in heterozygous form was two times higher in Sicilian patients with AMI than in healthy controls [11]. In that study, the M694V allele remained as a significant risk to develop AMI even after adjusting well-recognized risk factors for AMI. In contrast, no increased rate of the M694V allele, neither in homozygous nor in heterozygous form, was found in FMF patients with CVD compared to the control FMF patients without CVD in an Israel study [50]. However, that study was limited by its relatively small sample size (23 FMF patients with CVD). 

Our study has several limitations. The results may not be generalizable to all FMF amyloidosis patients since subjects with some traditional factors such as smoking, clinical history of CVD, overt diabetes mellitus, obesity (BMI > 30 kg/m^2^), and untreated hypertension were not included in our study. However, these factors would likely add to the risk of CVD overall. Many patients received a diagnosis of FMF and amyloidosis simultaneously, and thus, the time from symptom onset to development of amyloidosis could not be calculated. Reports of the kidney biopsies for amyloidosis were registered as positive or negative without activity and chronicity index. Genotyping was conducted with Strip Assay, which was used for detecting the variants just for known mutational hotspots (twelve), and thus, we cannot rule out undetected *MEFV* variants. We are aware that there are confounding factors such as physical activity status, diet, and family history that may affect the results of our study. Despite these limitations, our study has several strengths, including the relatively large patient group size and long follow-up duration for such a rare complication after the advent of biological therapies.

## 5. Conclusions

In conclusion, our data demonstrate that a genotype–phenotype association exists in terms of endothelial dysfunction and atherosclerosis in FMF patients with amyloidosis. This suggests that FMF overall is associated with increased CVD risk and that the risk may be proportional to the disease severity (since some mutations cause worse disease than others). To our knowledge, this is the first study that shows that FMF patients with amyloidosis and the M694V/M694V genotype are associated with higher risk factor measures, which might be predictive of worse clinical CVD outcomes, but that would need to be assessed in future studies. Longitudinal studies are needed to confirm these findings, namely the risks for future CVD events for patients with FMF.

## Figures and Tables

**Table 1:** Comparison of demographic and episode characteristics between FMF-related amyloidosis patients with (Group 1) and without (Group 2) homozygous M694V genotype.

	Total (*n* = 169)	Group 1 (*n* = 76)	Group 2 (*n* = 93)	
		Mean ± SD	Mean ± SD	Mean Difference [95% CI]
Age (years)	34.9 ± 5.8	35.5 ± 6.3	34.5 ± 5.3	0.9 [(−0.8)–(2.7)]
Age at FMF diagnosis (years)	15.5 ± 4.8	15.2 ± 4.7	15.8 ± 4.8	−0.5 [(−2.1)–(0.8)]
Age at amyloidosis diagnosis (years)	20.2 ± 2.8	20 ± 3.2	20 ± 2.5	−0.03 [(−0.9)–(0.8)]
Follow-up duration (months)	90 ± 8	88 ± 9	91 ± 7	−3.1 [(−5.6)–(−0.6)]
		***n* (%)**	***n* (%)**	***p***
Sex, male	104 (61.5)	50 (65.8)	54 (58.1)	0.30
Family history of FMF	56 (33.1)	30 (39.5)	26 (28.0)	0.11
Family history of amyloidosis	41 (24.2)	19 (25.0)	22 (23.7)	0.83
Fever	143 (84.6)	65 (85.5)	78 (83.9)	0.76
Abdominal pain	121 (71.6)	55 (72.4)	66 (71.0)	0.84
Arthritis	113 (66.9)	58 (76.3)	55 (59.1)	0.018
Chest pain	101 (59.8)	47 (61.8)	54 (58.1)	0.61
Arthralgia	81 (47.9)	39 (51.3)	42 (45.2)	0.42
Vomiting	52 (30.8)	26 (34.2)	26 (28.0)	0.38
Myalgia/myositis	46 (27.2)	20 (26.3)	26 (28.0)	0.81
Diarrhea	32 (18.9)	17 (22.4)	15 (16.1)	0.30
Protracted febrile myalgia	26 (15.4)	10 (13.2)	16 (17.2)	0.46
Fatigue	22 (13.0)	10 (13.2)	12 (12.9)	0.96
Headache	19 (11.2)	7 (9.2)	12 (12.9)	0.45
Erysipelas-like erythema	10 (5.9)	7 (9.2)	3 (3.2)	0.11 *
Treatment				
Colchicine	169 (100%)	76 (100%)	93 (100%)	
Anakinra	24 (14.2%)	8 (10.5%)	16 (17.2%)	0.22
Canakinumab	25 (14.7%)	9 (11.8%)	16 (17.2%)	0.33

* Fisher’s exact test.

**Table 2:** Comparison of the characteristics of FMF episodes between Group 1 and Group 2.

	Group 1 (*n* = 76)	Group 2 (*n* = 93)		
	Mean ± SD	Mean ± SD	Mean Difference [95% CI]	*p*
SBP (mm/hg)	130.9 ± 7.2	130.1 ± 7.6	0.84 [(−1.4)–(3.1)]	0.46
DBP (mm/hg)	86.1 ± 3.6	86.8 ± 4.7	−0.7 [(−1.9)–(0.6)]	0.30
BMI (kg/m^2^)	26.6 ± 2.2	26.6 ± 2.6	−0.04 [(−0.7)–(0.7)]	0.99
Cholesterol (mg/dL)	269.6 ± 59.1	271.2 ± 48.2	−1.6 [(−18.3)–(15)]	0.85
Triglyceride (mg/dL)	186.3 ± 54.9	197.9 ± 46.9	−11.7 [(−27.2)–(3.8)]	0.14
LDL (mg/dL)	151.9 ± 31.1	152.0 ± 28.1	−0.04 [(−9.1)–(8.9)]	0.99
HDL (mg/dL)	44.9 ± 6.3	45.3 ± 6.9	−0.4 [(−2.4)–(1.6)]	0.72
Glucose (mg/dL)	80.1 ± 14.3	81.9 ± 15.5	−1.8 [(−6.4)–(2.8)]	0.44
Insulin (μUI/mL)	13.8 ± 6.3	13.8 ± 5.9	−0.03 [(−1.9)–(1.8)]	0.97
HOMA	2.7 ± 1.4	2.8 ± 1.3	−0.03 [(−0.4)–(0.4)]	0.86
Ca (mg/dL)	9.1 ± 0.8	8.7 ± 0.6	0.4 [(0.2)–(0.6)]	<0.001
P (mg/dL)	4.8 ± 1.1	4.5 ± 1.0	0.3 [(−0.001)–(0.6)]	0.06
PTH (pg/dL)	63.4 ± 28.8	53.9 ± 21.2	9.5 [(1.6)–(17.3)]	0.02
25OHVitD (nmol/dL)	48.9 ± 12.9	49.9 ± 11.8	−1.02 [(−4.8)–(2.7)]	0.59
Albumin (g/dL)	3.4 ± 0.7	3.4 ± 0.7	−0.002 [(−0.2)–(0.2)]	0.98
GFR (mL/min/1.73 m^2^)	89.9 ± 7.2	90.0 ± 6.8	−0.1 [(−2.2)–(2.0)]	0.92
hsCRP (mg/dL)	21.3 ± 12.0	20.0 ± 12.6	1.3 [(−2.5)–(5.0)]	0.51

SBP, systolic blood pressure; DBP, diastolic blood pressure; BMI, body mass index; LDL, low-density lipoprotein; HDL, high-density lipoprotein; HOMA, homeostasis model assessment; PTH, parathyroid hormone; 25OHVD, 25 hydroxy-vitamin D; GFR, glomerular filtration rate; hsCRP, high-sensitivity C-reactive protein.

**Table 3:** Comparison of surrogate biomarkers for cardiovascular disease risk between 2 groups.

	Group 1 (*n* = 76)	Group 2 (*n* = 93)		
	Mean ± SD	Mean ± SD	Mean Difference [95% CI]	*p*
Proteinuria (g/24 h)	7966.3 ± 3526.4	4895.4 ± 1184.8	3070.9 [2230.9–3910.9]	<0.001
FMD (%)	5.85 ± 1.03	6.46 ± 0.83	−0.6 [(−0.89)–(−0.31)]	<0.001
cIMT	0.79 ± 0.15	0.67 ± 0.12	0.12 [0.08–0.16]	<0.001
FGF23 (pg/dL)	55.9 ± 26.2	43.2 ± 16.3	12.8 [5.9–19.6]	<0.001
PTX3	19.4 ± 17.6	6.1 ± 7.9	13.3 [8.9–17.5]	<0.001

FMD, flow-mediated dilatation; cIMT, carotid intima-media thickness; FGF23, fibroblast growth factor 23; PTX3, pentraxin-3.

**Table 4:** Correlation between parameters according to groups.

			FMD	cIMT	FGF23	PTX3	Proteinuria
Group 1	cIMT	r	−0.439				
		*p*	0.000				
	FGF23	r	−0.476	0.404			
		*p*	0.000	0.000			
	PTX3	r	−0.482	0.614	0.455		
		*p*	0.000	0.000	0.000		
	Proteinuria	r	−0.618	0.519	0.596	0.613	
		*p*	0.000	0.000	0.000	0.000	
	hsCRP	r	−0.269	0.205	0.395	0.090	0.360
		*p*	0.019	0.075	0.000	0.437	0.001
			**FMD**	**cIMT**	**FGF23**	**PTX3**	**Proteinuria**
Group 2	cIMT	r	0.099				
		*p*	0.346				
	FGF23	r	−0.313	0.215			
		*p*	0.002	0.039			
	PTX3	r	−0.128	0.432	0.329		
		*p*	0.220	0.000	0.001		
	Proteinuria	r	−0.460	−0.046	0.192	−0.070	
		*p*	0.000	0.663	0.066	0.504	
	hsCRP	r	−0.124	0.185	0.379	0.248	−0.002
		*p*	0.237	0.076	0.000	0.000	0.986

FMD, flow-mediated dilatation; cIMT, carotid intima-media thickness; FGF23, fibroblast growth factor 23; PTX3, pentraxin-3; hsCRP, high-sensitivity C-reactive protein.

## Data Availability

All data relevant to the study are included in the article. Requests to access the datasets should be directed to the author E.D.
